# Effects of a cluster randomized controlled kindergarten-based intervention trial on vegetable consumption among Norwegian 3–5-year-olds: the BRA-study

**DOI:** 10.1186/s12889-019-7436-3

**Published:** 2019-08-13

**Authors:** Anne Lene Kristiansen, Mona Bjelland, Anne Himberg-Sundet, Nanna Lien, René Holst, Lene Frost Andersen

**Affiliations:** 1 Department of Nutrition, Institute of Basic Medical Sciences, University of Oslo, PO Box 1046, Blindern, 0317 Oslo, Norway; 2 Department of Biostatistics, Institute of Basic Medical Sciences, University of Oslo, PO Box 1122, Blindern, 0317 Oslo, Norway

**Keywords:** Preschool children, Kindergarten-based intervention, Vegetables, Norway

## Abstract

**Background:**

Early childhood represents a critical period for the establishment of long-lasting healthy dietary habits. Limited knowledge exists on how to successfully increase vegetable consumption among preschool children. The overall aim of the present study was to improve vegetable intake among preschool children in a kindergarten-based randomized controlled trial.

**Methods:**

The target group was preschool children born in 2010 and 2011, attending public or private kindergartens in two counties in Norway. Data about child intake of vegetables were collected by three methods. First, parents filled in a web-based questionnaire of the child’s vegetable intake. Second, among a subsample, trained researchers observed children’s vegetable intake in the kindergarten. Thirdly, a parental web-based 24-h recall assessing the child’s vegetable intake was filled in. For allocation of kindergartens to intervention and control groups, a stratified block randomization was used. Multiple intervention components were implemented from September 2015 to February 2016 and components focused at influencing the four determinants availability, accessibility, encouragement and role modelling. The effect of the intervention from baseline (spring 2015) to follow-up 1 (spring 2016) was assessed by mixed-model analysis taking the clustering effect of kindergartens into account.

**Results:**

Parental consent was obtained for 38.8% of the children (633 out of 1631 eligible children). Based on the observational data in the kindergarten setting (n 218 in the control group and n 217 in the intervention group), a tendency to a small positive effect was seen as a mean difference of 13.3 g vegetables/day (95% CI: − 0.2, 26.9) (*P* = 0.054) was observed.

No significant overall effects were found for the total daily vegetable intake or for the parental reported frequency or variety in vegetable intake.

**Conclusions:**

Based on the observational data in the kindergarten setting, a tendency to a small positive effect was seen with a mean difference of about 13 g vegetables/day, while no other effects on child vegetable intake were found. Additionally, further research to understand the best strategies to involve parents in dietary interventions studies is warranted.

**Trial registration:**

International Standard Randomised Controlled Trials ISRCTN51962956. Registered 21 June 2016 (retrospectively registered).

**Electronic supplementary material:**

The online version of this article (10.1186/s12889-019-7436-3) contains supplementary material, which is available to authorized users.

## Introduction

A high intake of fruits and vegetables promotes health by reducing the future risk of several non-communicable diseases [1–4]. Even though numerous countries include intake of fruits and vegetables in their dietary guidelines, intake remains a challenge [5]. In Norway, the latest national dietary surveys among adults, school-aged children and preschool-aged children all report low consumption of vegetables [6–9]. Early childhood represents a critical period for the establishment of long-lasting healthy dietary habits as intake of fruits and vegetables may track from early years into later childhood or adulthood [10, 11].

Despite this awareness, limited knowledge exists on how to successfully increase vegetable consumption among preschool children. A Cochrane review of interventions aiming to increase consumption of fruit or vegetables or both among children aged five years and younger was recently updated [12]. This review included 63 studies and concluded that the evidence for *how* to increase children’s fruit and vegetable intake is limited [12]. A review by Hendrie et al. [13] included interventions aiming to increase vegetable intake in the home and in the community settings among children aged 2–12 years. Of the 22 included studies, only six studies were conducted in the preschool setting and none of these had a sole focus on increasing vegetable intake. Out of the six studies, four were reported to be effective in increasing vegetable intake in the short term (three months or less), while three were reported to be effective in increasing vegetable intake in the long term (six months or more) [13]. A systematic review by Holley and co-workers [14] concluded that repeated exposure was the most successful method of increasing vegetable consumption among children aged 2–5 years. Six out of seven studies on the effect of repeated exposure showed a significant increase in child vegetable consumption [14]. Finally, the systematic review by Nekitsing and co-workers [15] aimed to summarize strategies to increase vegetable intake among 2–5-year-old children. The review included 30 papers published between 2005 and 2016, and a meta-analysis showed that including taste exposure was the most effective method for increasing vegetable consumption [15]. Moreover, influencing environmental factors such as increased availability might be a promising way of increasing vegetable intake among children and adults [16, 17].

In Norway, more than 90% of all children aged 1–5 years attended kindergarten and most attend for 41 h and more per week [18]. National dietary guidelines recommend kindergartens to serve or provide food for at least three meals per day [19]. Research show that meals in Norwegian kindergartens are either brought from home (lunch box), provided by the kindergarten or a combination of the two [20]. As the main meal, most kindergartens provide a cold meal with bread. Additionally, most kindergartens provide fruits every day while only 36% of the kindergartens report to provide vegetables equally often [20].

From school-based interventions, there is evidence for multi-component interventions being the most effective in increasing intake of fruits and vegetables [21, 22]. As there have been conducted few multi-component intervention studies focusing on increasing vegetable intake among children of preschool age [12], the present study aimed to address this gap by developing, implementing and evaluating the effect of a multi-component intervention study conducted in the kindergarten and the home setting. This paper presents the effect of an intervention study on children’s vegetable intake from baseline to follow-up 1.

## Methods

### Study design and subjects

**Fig. 1:**
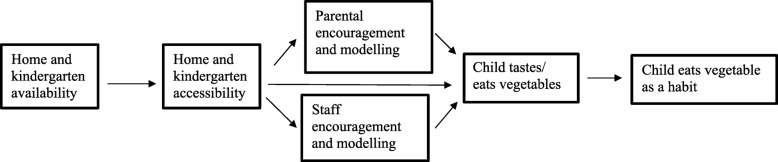
Model for change in determinants and vegetable intake in the BRA-study

The BRA-study (an acronym for the Norwegian words “Barnehage” (kindergarten), “gRønnsaker” (vegetables) and “fAmilie” (family)) is a cluster randomized controlled trial with an overall aim to improve vegetable intake (primary outcome) among preschool children (3–5 years at baseline) through changing the food environment and food-related practices in the kindergarten and the home (secondary outcomes). More specifically, the aim was to increase the daily frequency of vegetable intake, the variety of vegetables eaten over a month and the daily amount of vegetables consumed by changing the four determinants; availability, accessibility, encouragement and role modelling by kindergarten staff and parents (Fig. 1). Study subjects has previously been described [23]. In brief, target group for the current study was preschool children with year of birth 2010 and 2011, attending public or private kindergartens in the counties of Vestfold and Buskerud, Norway. All regular kindergartens (n 479) in the two counties were invited by letter followed-up by a phone call to inform about the study and to motivate for participation. The sample consisted of 73 kindergartens (response rate 15.2%). Parental consent was obtained for 38.8% of the children (633 out of 1631 eligible children). Number of participating children in each kindergarten varied from zero to 22 children, with a median of eight children per kindergarten. This study was conducted according to the guidelines laid down in the Declaration of Helsinki, and the Norwegian Center for Research Data approved all procedures involving human subjects. Data Protection Officer at the University of Oslo has assessed data privacy issues according to General Data Protection Regulations (GDPR).

### Detectable effect size

A sample size of 30 kindergartens with 10 children in each was estimated to be sufficient to detect a difference of 12 g in daily vegetable intake between the intervention and the control group. This was based on a standard deviation of 49 g vegetables per day [24] and an intra class (school) correlation coefficient of 0.15 [25] and assuming a significance level of 5% and a power of 80%.

### Randomization

**Fig. 2:**
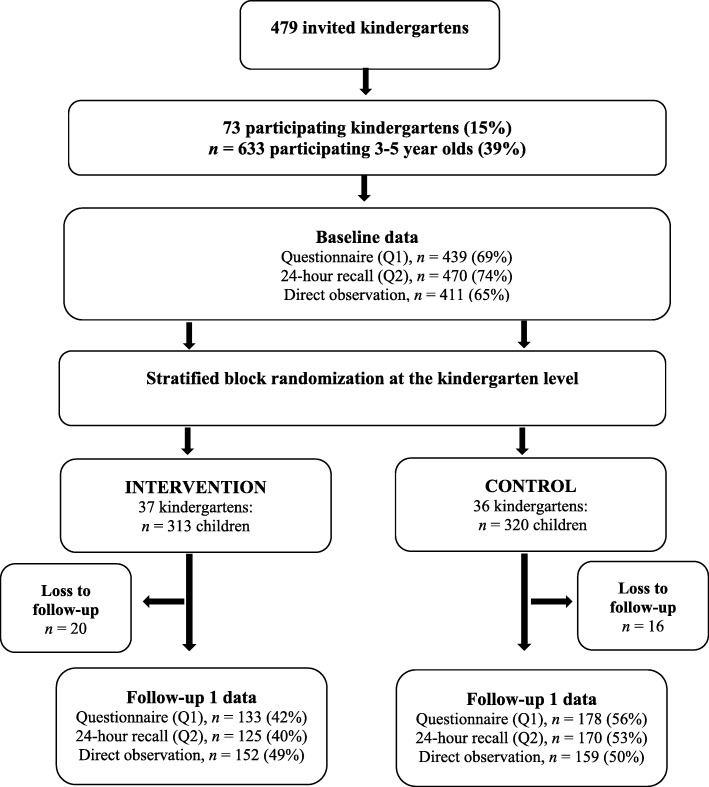
Flow diagram of recruitment, randomization, and participation of children in the BRA-study

An external statistician conducted a stratified block randomization. The randomization ensured an equal distribution of kindergartens within ownership (public and private) in the two groups and total number of participating children in each group. Hence, 320 children (51%) were allocated to the control group and 313 children (49%) were allocated to the intervention group (Fig. 2). Randomization was conducted after the baseline data collection.

### Intervention components

The intervention is described in accordance with the TIDieR guidelines [26] (Additional file 1). Additionally, the TIDieR checklist for cluster-randomized controlled trials have been completed (Additional file 2).

The intervention was implemented from September 2015 to February 2016 with one immediate evaluation (follow-up 1) and one follow-up evaluation one year after (follow-up 2). Multiple intervention components, presented in Additional file 1, were provided to improve children’s vegetable consumption both at home and in the kindergarten. The kindergartens were encouraged to implement and change their food practices in accordance with their own plans. Briefly, each kindergarten in the intervention group (n 37) was invited to a one-day inspirational course (kitchen practice and theory) in September 2015 where the intervention components were delivered. This course consisted of a brief introduction of the rationale for the study, a practical training in the kitchen making vegetable soup and vegetables with a dip in small groups under the instruction of a cook. The cook responsible for the kitchen practice observed and rehearsed the practical session developed by the Geitmyra culinary center for children. One principal investigator (NL), accompanied by either the post doc (ALK) or the PhD-student (AHS), conducted the theoretical part. The theoretical session went through research and practical ideas related to the four determinants availability, accessibility, encouragement and role modelling. At the end of the day, kindergartens started to make action plans where the content of the day was applied to the needs and possibilities of each kindergarten. At the inspirational day, each kindergarten received material for the kindergartens and a “Welcome package for the family” including the intervention components to the home. The material for the kindergartens consisted of aprons, a vegetable memory game, booklets, one hand blender (MQ 5007 Puree+ from Braun) per kindergarten, posters and brochures. The material for the families consisted of a cover letter explaining the rationale and the purpose of the intervention, a brochure, a stack of post-it’s and a booklet. The welcome package for the family was given to all parents/children in the kindergarten departments that attended the BRA-study, regardless of child participation in the BRA-study. The kindergarten staff was encouraged to find the best way to inform parents and hand out the package in their kindergarten; moreover, they were instructed to train/inform the relevant staff in their kindergarten. The implementation of the intervention by the kindergarten staff was supported by developing their own action plans within 4 weeks after the inspirational day. Moreover, a login protected website for the kindergartens and the parents containing all materials, as well as additional information about vegetables and the four determinants (availability, accessibility, role model and encouragement) was developed by the research team. Both kindergarten staff and parents were invited to attend a closed Facebook group developed for the study. In November 2015 and in February 2016 both kindergartens and the families received booster activities to maintain their focus on the intervention components in the home and in the kindergarten. Booster activities included booklets with recipes, a vegetable card/poster to register when and which vegetables were served for three days with potential for winning a gift card and suggestion of how to play tasting games with the children. Moreover, suggestions of activities to grow vegetables at three levels of difficulty and ideas for how to change the four determinants (availability, accessibility, role model and encouragement) were included. The control kindergartens and families continued as normal for the duration of the study and participated only by providing data, however they were offered access to the intervention website resources in September 2017.

### Data collection

A detailed description of the design of the study has been published previously [23]. In brief, at baseline and at follow-up 1, data about child intake of vegetables was collected by two parental web-based questionnaires assessing frequency, variety and amount. In addition, among a subsample the research team directly observed children’s vegetable intake at two meals in one day in the kindergarten. At baseline and at follow-up 1, those who returned two completed web-based questionnaires were entered in a lottery.

### Questionnaire assessing vegetable frequency and variety (Q1)

A link to a web-based questionnaire were sent out by e-mail to all parents of participating children (n 633 in 2015 and n 596 in 2016). By responding to the question “How often does your child eat the following vegetables?” parents reported frequency and variety of 18 different kinds of vegetables used by the child. Response alternatives were converted to times per day (times/day in parenthesis) “Never” (0), “1-3 times a month” (0.07), “1 time per week” (0.14), “2 times per week” (0.29), “3 times per week” (0.43), “4 times per week” (0.57), “5 times per week” (0.71), “6 times per week” (0.86), “every day” (1.0) and “2 or more times per day” (2.0). Potatoes, pickled and preserved vegetables were not included. Vegetable frequency was composed of adding up the use of the 18 vegetables to give the daily frequency and a vegetable was only defined as not used if the frequency was reported to be “never”. For vegetable variety, a vegetable was only defined as not used if the frequency was reported to be “never”. Hence, vegetable variety was composed of users of the 18 vegetables over a month and 18 was the maximum vegetable variety a child could have.

This questionnaire also requested information like child gender, child year of birth and maternal educational level (low (upper secondary school or less) and high (university college/university)).

### Questionnaire assessing vegetable amount (Q2)

All parents of participating children in the BRA-study received a link to a web-based 24-h recall by e-mail. The e-mail was sent out in the evening (after nine pm) on the day of the direct observation (see below). The 24-h recall was designed to measure the participating child’s intake of vegetables, fruits and berries in that day, with an extra focus on vegetable intake.

### Direct observation

Among a subsample, the research team directly observed children’s vegetable intake at two meals in one day in the kindergarten. All participating kindergartens (n 73) were visited by the research team in April–June 2015 and in April–June 2016. At follow-up 1 the aim was to observe the same children as those observed at baseline [23]. A detailed description of the design of the direct observation is published elsewhere [23].

Moreover, among another subsample (n 297), total daily vegetable amount was calculated by combining data from the direct observation when in kindergarten and the 24-h recall when at home (Q2). This was done by replacing the parentally reported intake of vegetables at lunch and at snack meal 2 in Q2 by the observed vegetable intake at these meals in the kindergarten.

### Data analysis

Data from baseline and follow-up 1 are included in this paper. Baseline differences between the intervention and the control groups were tested with chi-square tests and independent sample t-tests. The effect of the intervention was explored by mixed-models for intake of vegetables at follow-up 1 adjusted for baseline intake and using intervention/control as the primary covariate of interest. Kindergarten was used as a random effect to account for random inhomogeneity between kindergartens. All models were adjusted for child gender, child year of birth, maternal education level and kindergarten ownership (private or public). Participants were included in the analyses if they had data on either baseline and/or follow-up 1 and if they had data on all adjusting variables. The models were inspected by looking at the residuals and the Q-Q plots which indicated a roughly normal distribution of the data. Further, estimated marginal means were used to calculate the mean value for each of the two groups at baseline and at follow-up 1, adjusted for any other variable in the mixed-models.

All tests were two-sided with *P* values less than 0.05 considered statistically significant. All statistical analyses were performed by IBM® SPSS® Statistics, version 25.0 (IBM Corporation).

## Results

From baseline to follow-up 1, 16 children (5%) were lost to follow-up in the control group and 20 children (6%) were lost to follow-up in the intervention group (Fig. 2). The main reason for this loss was children moving to other kindergartens. Drop-out analysis showed no differences in background characteristics (child gender, child year of birth and maternal educational level) and kindergarten ownership between participating children (n 633) and those lost to follow-up (n 36) (data not shown).

Table 1 presents selected baseline characteristics of the children and the mothers (n 633). Boys and girls were equally represented in the two groups, as was child year of birth and maternal education. More children in the intervention group attended a public kindergarten compared to children in the control group (*P* < 0.001). Children in the intervention group had a significantly higher frequency (*P* = 0.009) and variety (*P* = 0.026) of vegetable intake at baseline compared to children in the control group. According to the total amount of vegetables consumed in one day at baseline, there were no significant differences between the groups (*P* = 0.102). However, for the direct observation, the results showed that children in the intervention group had a significantly higher intake of vegetables at baseline compared to children in the control group (*P* = 0.014).

**Table 1:** Baseline characteristics of 3–5-year-old children and their parents in the BRA-study (*n* 633)

	Control group *n 320*	Intervention group *n 313*	P^a^
Child gender			
Boy (%)	154 (48.1)	153 (48.9)	0.85
Girl (%)	166 (51.9)	160 (51.1)	
Child year of birth			
2010 (%)	167 (52.2)	164 (52.4)	0.96
2011 (%)	153 (47.8)	149 (47.6)	
Maternal education			
Low (Upper secondary school or less) (%)	86 (29.9)	92 (32.9)	0.44
High (University college/university) (%)	202 (70.1)	188 (67.1)	
*Missing (n)*	*32*	*33*	
Kindergarten ownership			
Public (%)	136 (42.5)	180 (57.5)	< 0.001
Private (%)	184 (57.5)	133 (42.5)	
Child vegetable intake			
Baseline frequency of vegetable intake (Q1)^b^ (times per day) (mean (SD)	2.9 (1.7)	3.3 (1.9)	0.009
Baseline variety in vegetable intake (Q1)^b^ (numbers per month) (mean (SD)	10.1 (4.4)	11.0 (4.1)	0.026
Baseline total vegetable amount (Q2 + direct observation)^c^ (grams per day) (mean (SD)	110.8 (86.2)	127.4 (80.0)	0.102
Observed baseline vegetable amount (direct observation only)^d^ (grams per day) (mean (SD)	40.0 (39.0)	51.2 (51.8)	0.014

^a^P = chi square test for categorical variables and independent sample t-test for continuous variables

^b^control *n* = 217, intervention *n* = 222

^c^control *n* = 142, intervention *n* = 129

^d^control *n* = 206, intervention *n* = 205

**Fig. 3:**
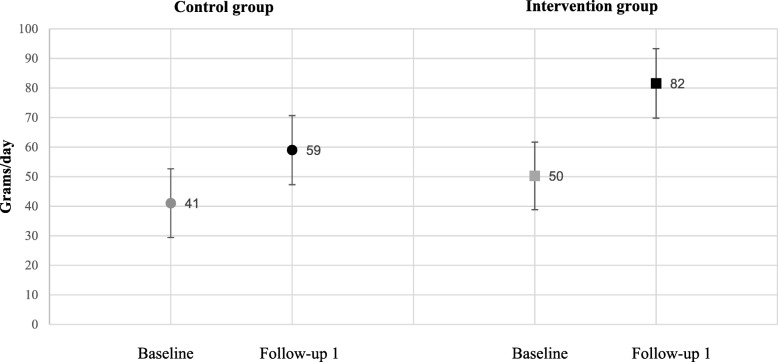
Changes in child vegetable intake from baseline to follow-up 1 by means of the direct observation when in kindergarten in the BRA-study (n 435)

Table 2 presents the adjusted effects of the BRA-intervention at follow-up 1. Mixed model analysis of the direct observation when in kindergarten showed a tendency to a small positive effect as a mean difference of 13.3 g vegetables per day (95% CI: − 0.2, 26.9) (*P* = 0.054) was observed. As shown in Fig. 3, vegetable intake in the intervention group increased from 50 g per day at baseline to 82 g per day at follow-up 1, while the corresponding numbers for the control group was an increase from 41 g per day at baseline to 59 g per day at follow-up 1. No significant effects of the intervention at follow-up 1 were observed for the daily frequency of vegetable intake, where a mean difference of 0.15 times per day (95% CI: − 0.2, 0.5) (*P* = 0.408) was observed (Table 2). In the intervention group, estimated marginal means showed that vegetable frequency increased from 3.3 times per day at baseline to 3.5 times per day at follow-up 1, while for the control group vegetable frequency increased from 2.9 times per day at baseline to 3.0 times per day at follow-up 1 (data not shown). For variety of vegetables eaten over a month, the effect of the intervention was practically zero as a mean difference of 0.09 different kinds of vegetables eaten over a month (95% CI -0.7, 0.9) (*P* = 0.820) was observed (Table 2). For the intervention group, estimated marginal means showed that vegetable variety increased from 10.9 kinds of vegetables eaten per month at baseline to 11.3 kinds eaten per month at follow-up 1. The corresponding numbers for the control group was an increase from 10.2 kinds of vegetables eaten per month at baseline to 10.5 kinds eaten per month at follow-up 1 (data not shown). Finally, no significant effect of the total daily vegetable intake was detected, where a mean difference of 10.0 g of vegetables per day (95% CI -19.6, 39.5) (*P* = 0.507) was observed (Table 2). Estimated marginal means showed that total vegetable intake in the intervention group increased from 132 g per day at baseline to 162 g at follow-up 1, while the corresponding numbers for the control group were an increase from 114 g to 134 g per day (data not shown). When exploring vegetable amount assessed only by the parental reported data in Q2 (n 447), no significant effect of the intervention was observed for the mixed-model analyses (*p* = 0.978) (data not shown).

**Table 2:** Intervention effects by mixed model analysis of the BRA-study on vegetable outcome at follow-up 1

	INTERVENTION EFFECT*
	Estimate (95% CI) P
Frequency of vegetable intake (times/day)^a^	0.15 (−0.2, 0.5) 0.408
Variety in vegetable intake (types/month)^a^	0.09 (−0.7, 0.9) 0.820
Total vegetable amount (grams/day)^b^	10.0 (− 19.6, 39.5) 0.507
Vegetable amount from the direct observation (grams/day)^c^	13.3 (−0.2, 26.9) 0.054

*Fixed effects parameter estimates. Adjusted for kindergarten clustering, time, child gender, child year of birth, maternal education and kindergarten ownership (private or public). Participants were included in the analyses if they had data on either baseline and/or follow-up 1 and if they had data on all adjusting variables

^a^Frequency and variety of vegetable intake was calculated based on data from Q1 (*n* = 229 in the control group and *n* = 218 in the intervention group)

^b^Total vegetable amount was calculated based on data from the direct observation when in kindergarten and the 24-h recall when at home (Q2) (*n* = 160 in the control group and *n* = 137 in the intervention group)

^c^Observed amount of vegetables consumed in kindergarten was based on the direct observation when in kindergarten (*n* = 218 in the control group and *n* = 217 in the intervention group)

## Discussion

Based on the observational data in the kindergarten setting, a tendency to a small positive effect was seen with a mean difference of about 13 g vegetables per day. However, there were some variations between subjects. Moreover, no significant overall effects were found for the total daily vegetable intake or for the parental reported frequency or variety in vegetable intake.

Research has shown that interventions delivered in the preschool setting have been more successful in increasing child vegetable intake compared to home-based settings [13]. This is in line with findings in the present study. Additionally, research on the effect of the BRA-intervention on vegetables served in the kindergarten setting did show favorable effects of the intervention in this setting (Himberg-Sundet et al., resubmitted). A likely reason for such findings might have been the provision of staff training as this has previously been associated with increased vegetable intake among children [13]. Moreover, such results might also be a consequence of differences in repeated exposure of vegetables in the two settings. One can speculate that repeatedly offering rejected vegetables in the preschool setting might be more tolerated by children in this setting compared to reoffering vegetables in the home setting as research indicate that the number of times a mother reoffers rejected vegetables is negatively associated with “mothers’ concern about their children’s mood and tantrums” [27]. Further, the intervention components in the present study focused on changing the four determinants availability, accessibility, encouragement and role modeling in the kindergarten and the home setting. Environmental strategies such as improved availability might be a promising way of increasing vegetable intake among children as 36 of 39 intervention studies with increased provision of vegetables, mostly conducted among children, showed a favorable intervention effect [16]. Such results are also supported by a previous study including baseline data from the BRA-study [23], which observed a strong and positive cross-sectional association between higher availability and accessibility in the home setting and child vegetable consumption.

The present study showed that the overall effect in child intake of vegetables in the home setting was weak. We encouraged the kindergarten staff to find the best way to inform parents and hand out the intervention components to the home, making it a sustainable model for larger scale implementation. However only indirectly involving parents, defined by Hingle et al. [28] as “provision of information that did not require a parental response”, is most often not associated with positive effects in dietary interventions among children [28]. A lack of positive effects of the intervention in the home setting might also be attributable to not knowing the best strategies to involve parents [29] or not reaching parents due to weak implementation [30]. As parents and childcare staff often have shared responsibility for the dietary intake of preschool children, further research to understand the best strategies to involve parents and developing sustainable models for larger scale implementation in dietary interventions is warranted.

Based on the observational data in the kindergarten setting, a tendency to a small positive effect was seen with a mean difference of about 13 g vegetables per day, corresponding to a daily increase of about one cherry tomato. Others have also reported limited effects on vegetable intake in intervention studies among children. The Cochrane review by Hodder et al. [12] included 39 studies assessing the effect of child-feeding practices, like repeated exposure to vegetables. A meta-analysis on the short-term effect (less than 12 months) indicated a mean difference in vegetable intake of about 3.5 g in favor of the intervention group. Another recent systematic review and meta-analyses, including 43 papers on effects of repeated exposure and conditions strategies for increasing vegetable linking and intake, also indicated that repeated exposure may have a small positive effect on child vegetable consumption [31]. Further, 14 studies assessed the effect of parent nutrition education on child fruit and vegetable intake, and the meta-analysis on the short-term effect did not show any significant effects on children’s fruit and vegetable intake [12]. Additionally, a meta-analysis on the short-term effect of multi-component interventions indicated that the interventions had “a very small effect on child consumption of fruit and vegetables, equivalent to an increase of 0.37 cups of fruit and vegetables per day” [12]. The calculated mean short-term (3 months) increase in vegetable intake among children aged 2–12 years was 29% (range − 20 to + 87%) for the 22 studies conducted in the home and in the community setting [13], corresponding to a daily increase of about 20–40 g vegetables [13]. However, even a small increase in vegetable intake, such as that observed in the present study, is considered important [32]. Foundation of dietary habits tends to begin in early childhood and these habits may be important for establishing long-lasting healthy dietary behaviors that may transmit into later childhood and possibly adulthood [10, 11]. However, more research to reach larger and long-term sustainable impacts on vegetable intake in childhood is needed.

### Methodological considerations

Contradictory to previous research among Norwegian preschool children [6, 8] baseline intake of vegetables in the present study was high. Using dietary assessment methodology with an extra focus on measuring vegetable intake might have contributed to this. This is consistent with an earlier study by Krebs-Smith et al. [33] who observed a higher reported intake of fruits and vegetables with an extended number of fruits and vegetables included in the food frequency questionnaire. Accuracy when recording dietary intake is also affected by measurement and reporting errors [34], however as the same methods were used at both time points in the present study, it is likely that such errors were in the same directions at both time points. Moreover, measuring diet among preschool children can be challenging. Using direct observation gave a detailed picture of vegetable intake in the kindergarten setting where parents are not reliable reporters [35–37]. Thus, it seems important for future dietary intervention studies to include high-quality dietary methodology like direct observation in the kindergarten setting.

Questions assessing vegetable intake in Q1 and Q2 was based on questions used in national dietary surveys among Norwegian 2- and 4-year-olds [6, 8] mapping total dietary intake. A validation study has been undertaken for the questionnaire used among 2-year olds [38], but not for the modified questions used in the present study. Prior to using Q1and Q2, a pilot study including ten mothers was undertaken. However, Q1 and Q2 have not been tested for reliability or validity resulting in not knowing which of our measures was most reliable and valid. In addition, different persons could have filled in the questionnaires at baseline and at follow-up 1, leading to a lack of consistency between measures. Hence, we cannot exclude the risk of imprecise datasets.

Researchers were not blinded to intervention group during data collection at follow-up 1. However, as there occasionally were options for the researchers to observe the same child/children during the direct observation of vegetable intake, intraclass correlation (ICC) as an estimate of inter-rater reliability between pairs of observers was calculated. The level of agreement between pairs of observers at follow-up 1 was 0.99 for the 37 children that were observed by two researchers. This agreement did not change when split into intervention (*n* 24, ICC = 0.99) and control groups (*n* 13, ICC = 0.98). This corresponds to the ICC found in the baseline data collection [23]. Hence, this might indicate an unbiased observed vegetable intake.

Randomization at the kindergarten level was successful, however at the individual level there was an unequal distribution of children in the intervention and control groups according to kindergarten ownership. Nevertheless, as the number of children in each group was large we do not think this has influenced our results or conclusions.

### Strengths and limitations

One strength of the present study was the sole focus on strategies to increase vegetable intake among preschool children. It is suggested that strategies to increase vegetable intake are likely to differ from those strategies necessary to achieve increased fruit intake [39] as consumption of these food groups is likely to occur in different contexts [40]. Another strength is the inclusion of three different measures of different aspects of vegetable intake, namely variety, frequency and amount. Targeting both home and kindergarten settings was also a strength of the present study as previous studies targeting multiple settings have been associated with positive intervention effects [13]. This study has some limitations that are important to take into consideration when interpreting the results. The participation rate among both kindergartens and parents were low and the level of parental education was high. A low participation rate is a threat to the generalizability of the obtained results; hence, our finding might not apply to all children in this age group. Further, it is also likely that there was considerable variation in how the intervention was implemented. Lastly, many factors may affect children’s intake of vegetables. We included some of these factors in our models, but there might be others that were not included.

## Conclusions

This study is one of a few conducted among preschool children with a sole focus on increasing vegetable intake in the kindergarten and the home setting. Based on the observational data in the kindergarten setting, a tendency towards a small positive effect was seen with a mean difference of about 13 g vegetables per day, while no other effects on child vegetable intake were found. Further research to understand the best strategies to involve parents in dietary interventions studies is warranted. In addition, as parents have limited ability to recall their child’s diet when in childcare, this study shows the importance of including high-quality dietary methodology in this setting.

## Additional files

Additional file 1: The BRA-study intervention description according to the TIDieR checklist. (DOCX 27 kb)

Additional file 2: The TIDieR (Template for Intervention Description and Replication) Checklist. (DOCX 32 kb)

## Data Availability

Data used and analyzed during the current study will be available from the corresponding author upon request, provided compliance with current legislation for application for data access in Norway.

## Abbreviations

ICC: Intraclass correlation
Q1: Questionnaire assessing vegetable frequency and variety
Q2: Questionnaire assessing vegetable amount
The BRA-study: An acronym for the Norwegian words “Barnehage” (kindergarten), “gRønnsaker” (vegetables) and “fAmilie” (family)
