# Supplementary material for: Effects of a cluster randomized controlled kindergarten-based intervention trial on vegetable consumption among Norwegian 3–5-year-olds: the BRA-study
Source: BMC Public Health. 2019 Aug 13;19:1098. doi: 10.1186/s12889-019-7436-3 (PMC6692927; doi:10.1186/s12889-019-7436-3)
Supplement: Supplementary file 1 — The BRA-study intervention description according to the TIDieR checklist. (DOCX 27 kb) [file 12889_2019_7436_MOESM1_ESM.docx]

**Additional file 1: The BRA-study intervention description according to the TIDieR checklist**

| **Item** | **Description** |
| --- | --- |
| ***Name (1)*** | The BRA-study  «Barnehage»=kindergarten, «gRønnsker» = vegetables, «fAmilie»=family  Kindergarten-based, family involved multi-component intervention to promote vegetable consumption among 3-5 year olds. |
| ***Why (2)*** | Vegetables are recommended as part of a health promoting diet. Yet, vegetable consumption in Norway is lower than the recommended amount for adults as well as children, and vegetables are primarily included in the dinner meal. There are no specific recommendation for daily amount of vegetables for young children in Norway. Hence, we chose to use a daily intake of 180 grams vegetables per day as the goal for child vegetable intake, which is about 70% of the adult recommendation. More than 90 % of Norwegian 1-5 year olds attend kindergartens, and normative dietary guidelines recommend that kindergartens facilitates two nutritional full meals per day (lunch and afternoon meal), in addition they arrange for children to eat their breakfast when they arrive in the morning. The food for the meals can be provided by the kindergarten, brought from home or a combination. Based on the literature, children’s taste for specific foods can be modified by repeated exposure, encouragement and positive role modelling by adults and peers. The intervention thus aimed at increasing the frequency, variation and amount of vegetables eaten by 3-5 year olds by changing the practices related to the four determinants; vegetable availability, accessibility, encouragement and role modelling by kindergarten staff and parents through a multi-component intervention. |
| ***What – Materials (3)*** | *1. Training of kindergarten staff*  A one-day inspirational course was conducted. This course consisted of a brief introduction of the rationale for the study, a practical training in the kitchen making vegetable soup and vegetables with a dip in small groups under the instruction of a cook based on the practice of Geitmyra culinary center for children. Participants eat the food for lunch together before a theoretical session going through research and practical ideas related to the four determinants. The day ended with starting to make action plans where the content of the day was applied to the needs and possibilities of each kindergarten. Finally, the supporting material was handed out (2 & 3).  *2. Welcome package for kindergarten staff at training*  A booklet with vegetable recipes describing practical ideas for how to cook food and involve children in the cooking based on the practice of Geitmyra culinary center for children.   - There was one large (70x100 cm) poster with photos of vegetables from the Norwegian information bureau for fruit and vegetables and 6 small posters (A4) made for the project; two on amounts of vegetables and four with ideas of “what to do” for each of the four determinants. - Aprons (4 per unit) and a fruit and vegetable memory game from the Norwegian information bureau for fruit and vegetables. - Brochures (folded A3) about the project, the ideas for how to change the four determinants, login instruction for the website and the Facebook group for all staff at the participating unit. - Information about the Norwegian national guidelines for food and meals in the kindergartens and the Norwegian dietary guidelines to all staff. - One hand blender (MQ 5007 Puree+ from Braun) per kindergarten.   *3. Welcome package for the parents*  A cover letter explaining the rationale and purpose of the intervention. A brochure with vegetable recipes and ideas for how to change the four determinants made for the project in collaboration with the Norwegian information bureau for fruit and vegetables. A stack of post-it’s with the slogan to be used to prompt buying and eating vegetables. A booklet to read for the child about “Mons and Mona shopping” made by the Norwegian information bureau for fruit and vegetables.  *4. Website with materials for kindergarten staff and parents*  Two login-protected websites (<https://www.med.uio.no/imb/forskning/prosjekter/bra-studien/ressurser/>) were made - one for staff and one for parents. The websites contained mostly the same information but were adapted when relevant to better fit the targeted setting. The website contained facts about vegetables, recipes of various level of difficulty, small articles (some with links to films) related to the four determinants, as well as a word-version and a pdf-version of all material given out during the intervention.  *5. Facebook group*  A closed Facebook group for both staff and parents were made for them to share ideas and discuss challenges.  *6. Booster activity 1 for kindergarten staff (November 2015)*  A booklet with recipes on cabbage, a vegetable card/poster to register when and which vegetables were served for three days with potential for winning a gift card by sending it to the project group, a sheet with suggestion of how to play tasting games with the kids.  *7. Booster activity 1 for parents (November 2015)*  An e-mail for the parents to be forwarded by staff with a letter with ideas and suggestion of how to play tasting games with the kids and a vegetable card for the individual child at home and promoting the potential for winning a gift card by sending it in same as for the kindergarten.  *8. Booster activity 2 for kindergarten staff (February 2016)*  A booklet with spring recipes (mixed salads and vegetable spread) and suggestions of activities to grow vegetables at three levels of difficulty.  *9. Booster activity 2 for parents (February 2016)*  A cover letter. A folded A3 sheet with all the ideas for how to change the four determinants. Five activity sheets to be completed with the child related to the same four factors. Two booklets of “Mons and Mona” (tasting and cooking vegetable soup) made by the Norwegian information bureau for fruit and vegetables and a stack of post-it’s with the slogan. |
| ***What - Procedures (4)*** | The kindergarten staff was trained and provided with the material and the welcome package for the parents at a one-day inspirational course. They were instructed to train/inform the relevant staff of their kindergartens, submit an action plan within 4 weeks after they attended the course and deliver the Welcome package to the parents as bested fitted their activities, but within a reasonable amount of time.  Booster activities were sent to the kindergartens at two and five months after the training course. The family components were distributed through the kindergartens. |
| ***Who provided (5)*** | At least one person from each kindergarten was trained by the research team (theory) and a cock (kitchen practice) in a one-day inspirational course. The four kindergartens who did not attend the course were visited by the research team and given a 1.5-2 hour theoretical introduction to the intervention and provided with the Welcome package for kindergarten and the Welcome package for parents. The kindergarten person trained could belong to any of these categories: Kindergarten assistant; no formal education required, Pedagogical leader; three years of formal education as a kindergarten teacher (bachelor degree), Kindergarten leader; three years of formal education as a kindergarten teacher (bachelor degree) or other similar education, Cook/the one responsible for the food served in the kindergarten; no formal education. |
| ***How (6)*** | The staff training was conducted face-to-face and the kindergarten staff was provided with the power-point presentation including the manuscript through the project’s website.  The intervention was delivered by the kindergarten staff to the children as part of the daily meals and any additional pedagogical activities conducted in the participating department in the intervention kindergartens. Each department had between 12-25 children aged 3-5 years and usually employed 3-4 staff.  The intervention to the families was delivered by print or e-mail to the parents by the kindergarten staff. The kindergarten staff could also address the intervention topic face-to-face during daily delivery and pick-up routines or at parent meetings/events. |
| ***Where (7)*** | The staff training was conducted at three different locations to allow easy access for the participants. One was a fully equipped teaching kitchen at a university college, while the two others were kitchens used for home economics at two primary schools.  The intervention itself was delivered in the 37 intervention kindergartens within the physical, economic and staffing resources available to each of them and the usual communication routines with parents. |
| ***When and how much (8)*** | The intervention was aimed at changing availability and vegetable-related practices in the kindergartens and homes and was implemented from September to February the following year. The kindergartens and parents choose themselves how to work with the intervention material. |
| ***Tailoring (9)*** | The kindergarten staff was instructed at the training to apply what they had learned to their local situation when making their action plan. The parents were encouraged to use the strategies that best fitted their child and their families in eating situations, and make small, gradual changes rather than large ones. |
| ***Modifications (10)*** | The intervention was not modified during the course of the study. |
| ***How well –***  ***Planned (11)*** | Process evaluation on perceptions and use of the intervention components were collected from the staff participating on the inspirational day training and midway through the first year of the intervention (January 2016) through online questionnaires to staff and parents. Selected kindergartens who reported many (mean n=31) or few (mean n=20) practices were interviewed to understand the differences. |
| ***How well - Actual (12)*** | The process evaluation showed that the staff was initially positive to the training and the intervention components, but the usefulness of the components were scored lower half way through the first intervention year than immediately after the training. Components involving the children were more highly rated and kindergartens with previous experience in diet related projects made more changes to their practices.  The response rate of the parents was low and a higher proportion reported that they did not see a need to change their practices. |
